# Small Cell Size Circulating Aneuploid Cells as a Biomarker of Prognosis in Resectable Non-Small Cell Lung Cancer

**DOI:** 10.3389/fonc.2021.590952

**Published:** 2021-03-03

**Authors:** Yang Hong, Jiahui Si, Jie Zhang, Ying Xiong, Jianzhi Zhang, Peter Ping Lin, Jian Fang, Yue Yang, Chao Lv, Yuanyuan Ma

**Affiliations:** ^1^Department of Thoracic Surgery II, Key Laboratory of Carcinogenesis and Translational Research (Ministry of Education), Peking University Cancer Hospital and Institute, Beijing, China; ^2^Department of Thoracic Oncology II, Key Laboratory of Carcinogenesis and Translational Research (Ministry of Education), Peking University Cancer Hospital and Institute, Beijing, China; ^3^Department of Oncology, Cytelligen, San Diego, CA, United States

**Keywords:** non-small cell lung cancer, circulating aneuploid cells, prognosis, resection, biomarker

## Abstract

**Objective:**

The size distribution of circulating aneuploid cells (CACs) and its clinical significance were investigated in resectable non-small cell lung cancer (NSCLC).

**Patients and Methods:**

A total of 50 patients with resectable NSCLC were enrolled in this study. Blood samples (50 pre-surgery and 35 post-surgery) were collected and used for the detection of CAC chromosome 8 heteroploidy through the subtraction enrichment and immunostaining fluorescence *in situ* hybridization (SE-iFISH) method.

**Results:**

Less than 20% small cell size and more than 80% large cell size CACs were detected. Karyotypes, including triploid, tetraploid, and multiploid, had varying distributions. The triploid subtype accounted for the majority of small cell size CACs, whereas the multiploid subtype accounted for the majority of large cell size CACs. We found that total small cell size and triploid small cell size CACs, but not large cell size CACs, derived from pre-surgery samples, were associated with shorter disease-free survival. Moreover, total small cell size and triploid small cell size CACs were associated with higher TNM stage and recurrence. Nevertheless, the variation between pre- and post-surgery CACs was not related to survival among patients with resectable NSCLC.

**Conclusions:**

Pre-surgery small cell size CACs, especially the triploid subtype, could be regarded as a potential prognostic biomarker for patients with resectable NSCLC.

## Introduction

Lung cancer is the leading cause of cancer-associated death worldwide (1). Non-small cell lung cancer (NSCLC) accounts for 85% of all lung cancer cases, with a low 5-year survival rate of less than 20% (2–4). A total of 45% of early-stage NSCLC patients experience postoperative recurrence, including those who have already undergone primary tumor resection (5). Therefore, biomarkers for predicting relapse in patients with resectable tumors are extremely important for dynamic clinical evaluation and treatment choice. Lower airway bacterial microbiomes, tumorspheres, circulating cell-free DNA, urine cell-free DNA, and circulating non-hematopoietic cells such as circulating tumor cells (CTCs), circulating endothelial cells (CECs), or circulating aneuploid cells (CACs) could reportedly be used as biomarkers for NSCLC diagnosis, relapse prediction, and drug resistance evaluation (6–11).

CTCs, CECs, and CACs are well-known for their association with metastasis and progression and provide information for individual therapy and prediction of prognosis in several cancer types such as breast, colorectal, prostate, and lung (7, 12–17). Epithelial markers such as EpCAM and cytokeratin are often used to enrich CTCs (18). However, this approach may miss an aggressive and clinically relevant subpopulation of tumor cells, partially because of the epithelial-mesenchymal transition (EMT) that occurs with a reduction in or loss of epithelial markers in the tumors (19, 20). This kind of EMT^+^ CTC is helpful in predicting poor outcome and managing therapy, which is important for patients (19). In addition, CTCs are a heterogeneous population; hence, clarification of its characteristics can shed light on tumor heterogeneity, recurrence mechanism, treatment efficacy, or poor prognosis for cancer patients (21, 22).

In addition to the numbers of CTCs, CECs, and CACs, the specific characteristics of these cells seem to be more significant as biomarkers. Aneuploidy is a common trait in solid tumors (23, 24); moreover, chromosomal redistribution contributes to proliferation during the evolution of tumor cells (25, 26). Examination of aneuploidy in chromosome 8 can identify CTCs in epithelial and glioma tumors with high sensitivity (20, 27, 28). Moreover, chromosomal instability status can reveal the heterogeneous phenotype of CTCs, CECs, and CACs (21). Li et al. suggested that an increased percentage of triploid CTCs was associated with chemotherapy resistance in advanced gastric cancer (27). Moreover, quantified chromosome ploidy may be a predictor of therapeutic efficacy and disease progression (29).

Herein, we analyzed the clinical significance of CACs and their aneuploidy subtypes in samples of peripheral blood from patients with resectable NSCLC. We found that small cell size CACs, especially the triploid small cell size CAC subtype, were correlated with the prognosis of patients with resectable NSCLC.

## Materials and Methods

### Patient Enrollment and Specimen Collection

A total of 50 patients who were newly diagnosed with NSCLC between December 2014 and December 2015 and received R0 resection at Peking University Cancer Hospital were enrolled in this study. These patients were histologically confirmed as having stage I to IIIA NSCLC, including 30 cases of adenocarcinoma, 18 of squamous carcinoma, and two of large cell lung cancer (**Table 1**). Peripheral blood samples (7.5mL) were collected from all patients before the surgery and again from 35 patients one week after resection. All blood samples were processed within 24 h of collection. Each patient provided written informed consent, and the Institutional Ethics Committee of Peking University Cancer Hospital approved this study. The study was conducted according to the principles of the Declaration of Helsinki.

**Table 1:** Characteristics of patients (n = 50).

	Variable	No. of cases	Percentage (%)
**Age**	≤60	16	32
	>60	34	68
**Gender**	Male	28	56
	Female	22	44
**Smoking**	No	25	50
	Yes	25	50
**Histology**	Adenocarcinoma	30	60
	Squamous carcinoma	18	36
	Large cell lung cancer	2	4
**Pre-treatment clinical TNM stage**	I	22	44
	II	3	6
	IIIA	25	50
**Pathological TNM stage**	I	28	56
	II	12	24
	IIIA	10	20
**T stage**	T1	18	36
	T2	27	54
	T3	5	10
**Lymph node station**	N0	31	62
	N1	10	20
	N2	9	18
**Lymph-vascular invasion**	No	46	92
	Yes	4	8
**Recurrence**	No	34	68
	Yes	16	32
**DFS range (months)**	0–12	5	10
	12–24	6	12
	24–36	7	14
	36–48	26	52
	48–60	6	12

### CAC Detection by SE-iFISH

To identify CACs, we performed subtraction enrichment and immunostaining fluorescence *in situ* hybridization (SE-iFISH) on the samples as previously described (30, 31). CAC enrichment was performed using the subtraction enrichment method. A 7.5mL blood sample from each patient was centrifuged at 600 × *g* for 5 min to separate the plasma. The sedimented cells were placed on top of 3 mL of anon-hematopoietic cell separation matrix (Cytelligen, San Diego, CA, USA) and then centrifuged at 400 × *g* for 5 min to deplete the red blood cells. To separate the leukocytes, immune-magnetic particles conjugated with anti-CD45 monoclonal antibodies were added and incubated with the supernatant obtained above at 25°C for 15 min. Next, the entire solution was added on the top of separation matrix again, followed by centrifuging at 400 × *g* for 5 min. Next, the supernatants were collected from above the magnetic beads and magnetic separation was performed; then, the bead-free solution was centrifuged again at 500 × *g* for 2 min. The cell pellet was mixed with 100 μL of cell fixative, then applied to the CAC slides. These slides underwent air-drying and were then suitable for iFISH.

Next, we performed iFISH on the resulting samples according to the kit’s instructions (Cytelligen). Prepared samples on the coated slides were hybridized for 4 h with the Vysis Centromere Probe (CEP8) Spectrum Orange (Abbott Laboratories, Abbott Park, IL, USA), followed by incubation with Alexa Fluor 594-conjugated monoclonal anti-CD45 antibodies (Cytelligen) at room temperature for 30 min. Finally, we used 4-6-diamidino-2-phenylindole (DAPI) (Life Technologies, Carlsbad, CA, USA) to stain the nuclei. At least two pathologists performed CAC counting for DAPI^+^ and CD45^-^ cells, identified chromosome 8 aneuploidy under fluorescence, and calculated cell size. CACs of ≤ 5 µm (approximately the size of a WBC or less) were considered small cell size CACs, whereas those>5 µm were considered large cell size CACs.

### Statistical Analyses

All statistical analyses were performed using IBM SPSS Statistics software version 23.0. Correlations of CACs with clinical or pathological characteristics were calculated and analyzed using the chi-square test or Fisher’s exact test, and logistic proportional hazards regression analysis was further used to analyze the multivariate hazard ratios. Disease-free survival (DFS) was defined as the duration from surgery to cancer relapse. Kaplan-Meier survival plots for 3-year DFS were generated based on whether patients were positive or negative for CACs pre- and post-surgery, and the log-rank test was used to compare survival curves. P < 0.05 was considered statistically significant. All P values were two-sided.

## Results

### Patient Characteristics

This study included 50 cases of NSCLC, of which 28 patients were male and 22 were female. The patients had a median age of 62 years and an average age of 61.5 years (range 39–81). Patient characteristics are presented in **Table 1**. For the pre-treatment clinical stage, 22 (44%), 3 (6%), and 25 (50%) patients were at stage I, II, and IIIA, respectively. In contrast, the numbers of patients at pathological TNM stages I, II, and IIIA were 28 (56%), 12 (24%), and 10 (20%), respectively. Pathological examination confirmed that 18 (36%), 27 (54%), and 5 (10%) patients were diagnosed with T1, T2, and T3 stages, respectively. In addition, 31 patients were diagnosed without lymph node metastasis (N0 stage), and 10 and 9 patients were diagnosed with N1 and N2 stages with lymph node metastasis, respectively. Among the 50 cases, four (8%) had lymph-vascular invasion. As for the follow-up data (DFS data are also shown in **Table 1**), tumor recurrence and progression occurred in 16 (32%) patients, whereas the status of the 34 (68%) other patients remained unchanged until the time of reporting.

### CAC Detection

**Figure 1:**
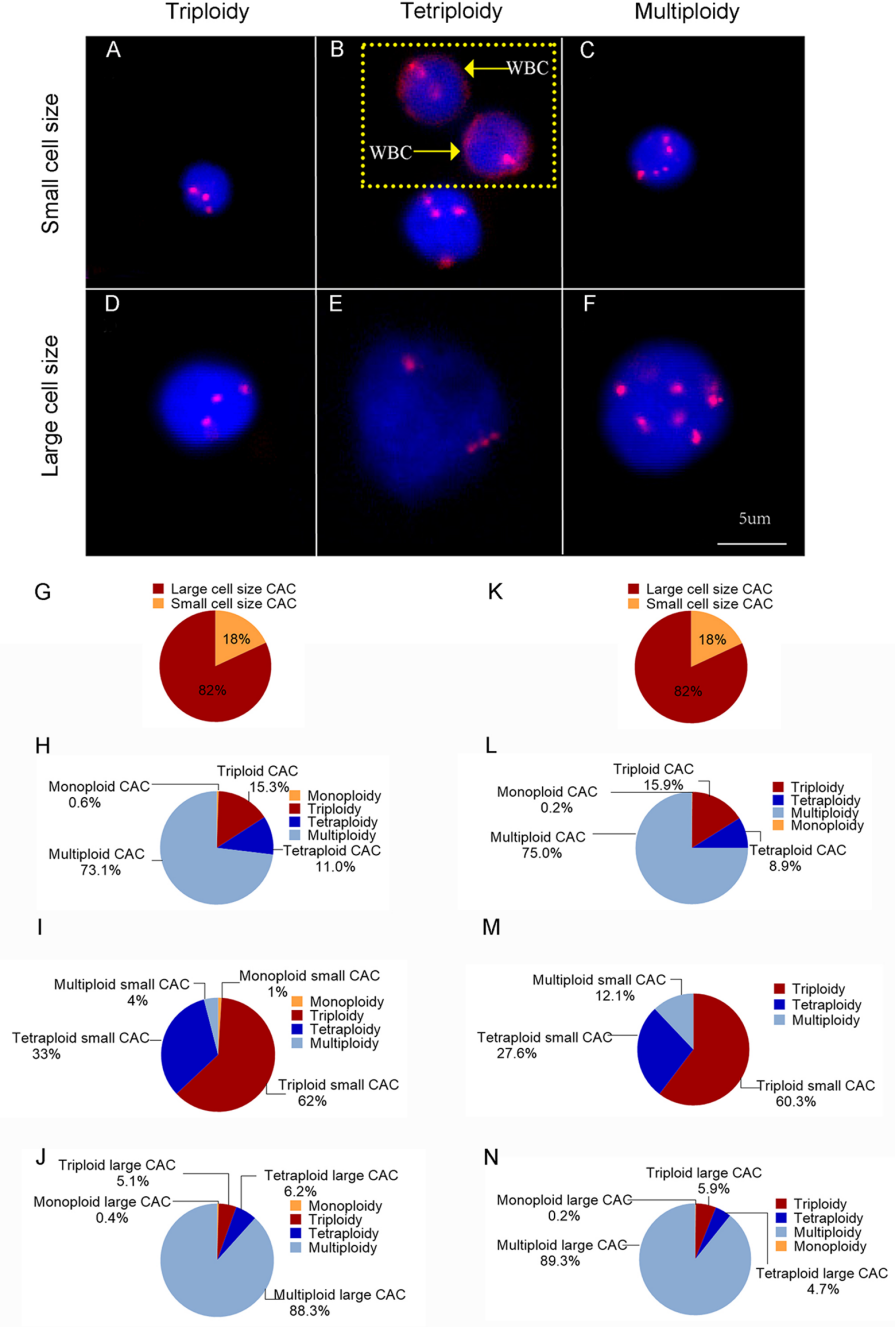
CAC detection **(A–F)** Representative images of circulating aneuploid cells (CACs). **(A–C)** Small cell size CAC. **(A)**Triploid small cell size CAC. **(B)** Tetraploid small cell size CAC. The WBC (CD45^+^) is indicated by a yellow arrow. **(C)** Multiploid small cell size CAC. **(D–F)** Large cell size CAC. **(D)**Triploid large cell size CAC. **(E)** Tetraploid large cell size CAC. **(F)** Multiploid large cell size CAC. **(G–J)** Pre-surgery CAC subtype distribution in 50 patients. **(G)** Proportion of small cell size and large cell size CACs. **(H)** Proportion of heteroploid subtypes of total CACs. **(I)** Proportion of heteroploid subtypes of small cell size CACs. **(J)** Proportion of heteroploid subtypes of large cell size CACs. **(K–N)** Post-surgery CAC subtype distribution in 35 patients. **(K)** Proportion of small cell size and large cell size CACs. **(L)** Proportion of heteroploid subtypes of total CACs. **(M)** Proportion of heteroploid subtypes of small cell size CACs. **(N)** Proportion of heteroploid subtypes of large cell size CACs.

CACs were identified as having an abnormal chromosome 8 karyotype (**Figure 1**). With the general size of WBCs as the threshold, CACs were identified as either small (≤ 5 µm) (**Figures 1A–C**) or large (>5 µm) (**Figures 1D–F**). As in previous studies (32, 33), CACs were further divided into triploid, tetraploid, and multiploid subtypes.

First, we analyzed the number and characteristics of CACs derived before surgery from 50 patients with resectable NSCLC. We found 554 CACs in our patient cohort, including 454 (82%) large cell size CACs and 100 (18%) small cell size CACs (**Figure 1G**). The heteroploid features were as shown in **Figures 1H–J**. The total and large cell size CACs contained more multiploid (73% and 88%) than triploid (15% and 5%) or tetraploid cells (11% and 6%). However, small cell size CACs had the largest proportion of triploid cells (62%), compared to tetraploid (33%) and multiploid cells (4%) (**Figures 1H–J**). In addition, total, small cell size, and large cell size CACs were detected in 88% (44/50), 66% (33/50), and 86% (43/50) of patients, respectively **(****Table 2**). There were 62%, 60%, and 82% positive rates for triploidy, tetraploidy, and multiploidy in the total CACs, respectively. For small cell size CACs, 56%, 44%, and 8% of patients had triploidy, tetraploidy, and multiploidy, respectively. For large cell size CACs, 22%, 34%, and 82% of patients had triploidy, tetraploidy, and multiploidy, respectively (**Table 2**).

**Table 2:** CAC detection before and after the surgery.

Variation	Pre-surgery (n = 50)			Post-surgery (n=35)		
	No. of CAC cases (%)	No. of small cell size CAC cases (%)	No. of large cell size CAC cases (%)	No. of CAC cases (%)	No. of small cell size CAC cases (%)	No. of large cell size CAC cases (%)
**Monoploidy**	1 (2%)	1 (2%)	1 (2%)	1 (3%)	0 (0%)	1 (3%)
**Triploidy**	31(62%)	28(56%)	11 (22%)	28 (80%)	25 (71%)	10 (29%)
**Tetraploidy**	30(60%)	22(44%)	17 (34%)	21 (60%)	12 (34%)	14 (40%)
**Multiploidy**	41(82%)	4 (8%)	41 (82%)	32 (91%)	8 (23%)	32 (91%)
**Total**	44(88%)	33(66%)	43 (86%)	33 (94%)	26 (74%)	33 (94%)

Next, we detected 628 CACs derived from 35 patients post-surgery. Compared to the proportion of CACs in pre-surgery samples, the same percentages of large cell size CACs (82%, 512) and small cell size CACs (18%, 116) were observed in post-surgery samples (**Figure 1K**). We observed a similar distribution of the different CAC heteroploidies between the pre-treatment and post-surgery samples (**Figures 1L–N**). Multiploid CACs were the major subtype among the total and large cell size CACs, with proportions of 75% and 89%, respectively, whereas the triploid subtype was the main component (60%) among small cell size CACs (**Figures 1L–N**). Furthermore, the positive rates were 94%, 74%, and 94% for total, small cell size, and large cell size CACs, with rates for triploidy, tetraploidy, and multiploidy similar to those detected pre-surgery (**Table 2**).

### CAC Subtypes and DFS

**Figure 2:**
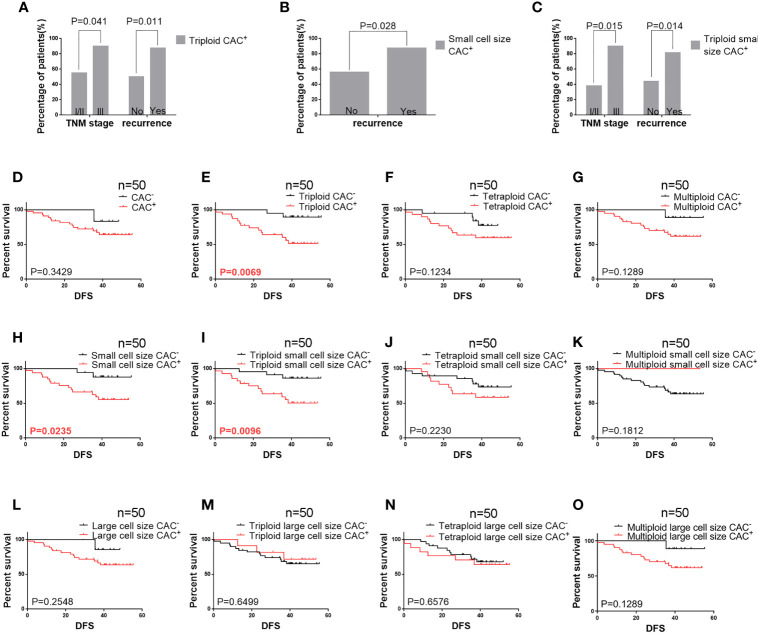
Pre-surgery CAC analysis in 50 patients **(A)** Correlation of pre-surgery triploid circulating aneuploid cells (CACs) with TNM stage and recurrence. The proportion of patients who had triploid CACs was higher among those with TNM stage III and recurrence than those with TNM stage I/II and without recurrence, respectively (P < 0.05). **(B)** Correlation between pre-surgery small cell size CACs and recurrence. The proportion of patients who had small cell size CACs was higher among those with recurrence than those without (P < 0.05). **(C)** Correlation of pre-surgery triploid small cell size CACs with TNM stage and recurrence. The proportion of patients who had triploid small cell size CACs was higher among those with TNM stage III and recurrence than those with TNM stage I/II and without recurrence, respectively (P < 0.05). **(D–O)** Survival analysis. **(D)** Total CAC was not correlated to DFS (P > 0.05). **(E)** Patients with triploid CACs had shorter DFS compared to those without triploid CACs (P < 0.05). **(F, G)** Tetraploid and multiploid CAC subtypes were not correlated to DFS (P > 0.05). **(H)** Patients with small cell size CACs had shorter DFS compared to those without (P < 0.05). **(I)** Patients with triploid small cell size CACs had shorter DFS compared to those without (P < 0.05). **(J, K)** Tetraploid and multiploid small cell size CAC subtypes were not correlated to DFS (P > 0.05). **(L–O)** Total large cell size CACs and triploid, tetraploid, and multiploid large cell size CAC subtypes were not correlated to DFS (P > 0.05).

We analyzed the correlations between histoclinical characteristics and pre-surgery CACs among our patients (**Table 3**). Unsurprisingly, triploid CACs were related to later-stage TNM and recurrence (p < 0.05). Moreover, 90% of TNM stage III patients had triploid CACs, whereas 55% of TNM stage I/II patients had triploid CACs (**Figure 2A**, left panel). Triploid CACs were present in 88% of patients who had relapsed and 50% of patients who had not relapsed (**Figure 2A**, right panel). More importantly, small cell size CACs were associated with a higher recurrence rate and were present in 88% of patients who had relapsed and 56% of patients who had not relapsed (**Figure 2B**). Furthermore, 90% of TNM stage III patients had triploid small cell size CACs; however, only 38% of TNM stage I/II patients had triploid small cell size CACs (**Figure 2C**, left panel). Triploid small cell size CACs were present in 81% of patients who had relapsed, and in 44% of patients who had not relapsed (**Figure 2C**, right panel). Nevertheless, there was no significant correlation between clinical features and large cell size CACs (**Table 3**). The multivariate hazard ratios for CACs are shown in **Table 4**. Recurrence was an independent predictive factor for positive triploid CACs, small cell size CACs, and triploid small cell size CACs; however, gender, age, and TNM were not independent predictive factors for positive CACs.

**Table 3:** Correlation between pre-surgery CAC and clinical characteristics (n = 50).

Variables	CAC								Small cell size CAC								Large cell size CAC							
	total		Triploidy		Tetraploidy		Multiploidy		total		Triploidy		Tetraploidy		Multiploidy		total		Triploidy		Tetraploidy		Multiploidy	
	+	*p*	+	*p*	+	*p*	+	*p*	+	*p*	+	*p*	+	*p*	+	*p*	+	*p*	+	*p*	+	*p*	+	*p*
**Gender**																								
Male (n=28)	25	0.752	19	0.336	20	0.063	23	0.976	21	0.130	17	0.449	14	0.335	2	0.801	25	0.450	6	0.912	12	0.136	23	0.976
Female (n=22)	19		12		10		18		12		11		8		2		18		5		5		18	
**Age**																								
≤60 (n=16)	15	0.391	11	0.500	12	0.137	12	0.377	12	0.357	9	0.981	8	0.558	0	0.153	14	0.834	4	0.725	8	0.101	12	0.377
>60 (n=34)	29		20		18		29		21		19		14		4		29		7		9		29	
**Smoking history**																								
Never (n=25)	21	0.384	13	0.145	12	0.083	20	0.713	14	0.136	12	0.254	10	0.569	2	1.000	20	0.221	6	0.733	6	0.136	20	0.713
Yes (n=25)	23		18		18		21		19		16		12		2		23		5		11		21	
**Histology**																								
ADC (n=30)	26	0.845	17	0.527	15	0.157	24	0.763	19	0.728	14	0.222	12	0.254	1	0.235	25	0.731	8	0.537	8	0.402	24	0.763
SCC (n=18)	16		13		14		15		13		13		10		3		16		3		8		15	
LCC (n=2)	2		1		1		2		1		1		0		0		2		0		1		2	
**TNM classification**																								
I/II (n=40)	34	0.192	22	**0.041**	22	0.149	32	0.462	24	0.073	19	**0.015**	17	0.669	3	0.794	33	0.154	10	0.306	13	0.654	32	0.462
III (n=10)	10		9		8		9		9		9		5		1		10		1		4		9	
**Recurrence**																								
No (n=34)	29	0.391	17	**0.011**	18	0.137	26	0.138	19	**0.028**	15	**0.014**	13	0.231	4	0.153	28	0.279	8	0.704	11	0.720	26	0.138
Yes (n=16)	15		14		12		15		14		13		9		0		15		3		6		15	

The bold values were P values less than 0.05, which were considered statistically significant.

**Table 4:** Logistic regression analysis of CAC and clinicopathologic characteristics among 50 patients with resectable NSCLC.

Variable	Triploid CAC			Small CAC			Triploid small CAC		
	Positive case number	Multivariate analysis		Positive case number	Multivariate analysis		Positive case number	Multivariate analysis	
		HR	P		HR	P		HR	P
**Gender**									
Male (n=28)	19	–	0.432	21	–	0.166	17	–	0.567
Female (n=22)	12			12			11		
**Age (year)**									
≤60 (n=15)	11	–	0.611	12	–	0.428	9	–	0.85
>60 (n=35)	20			21			19		
**TNM Stage**									
I/II (n=40)	22	–	0.193	24	–	0.254	19	–	0.078
IIIA (n=10)	9			9			9		
**Recurrence**									
No	17	7	**0.019**	19	5.526	**0.04**	15	5.489	**0.019**
Yes	14			14			13		

The bold values were P values less than 0.05, which were considered statistically significant.

**Figure 3:**
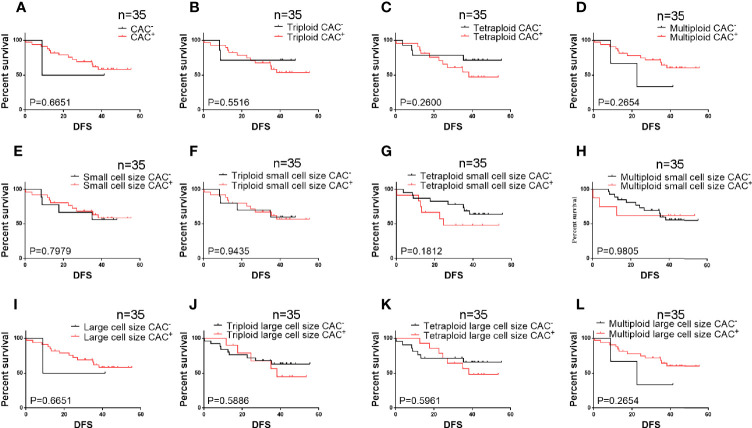
Survival analysis of post-surgery CACs among 35 patients **(A–D)** Total circulating aneuploid cells (CACs) and triploid, tetraploid, and multiploid CAC subtypes were not correlated to DFS (P > 0.05). **(E–H)** Total small cell size CACs and triploid, tetraploid, and multiploid small cell size CAC subtypes were not correlated to DFS (P > 0.05). **(I–L)** Total large cell size CACs and triploid, tetraploid, and multiploid large cell size CAC subtypes were not correlated to DFS (P > 0.05).

The relationship between DFS and total, small cell size, and large cell size CACs was further clarified (**Figures 2D–O**). Among these analyses, we found that pre-surgery triploid CACs, small cell size CACs, and triploid small cell size CACs were associated with shorter DFS (**Figures 2E, H, I**). However, the Kaplan-Meier curves showed no significant differences between the other CAC subtypes and outcome in NSCLC (**Figures 2D, F, G, J–O**). We also found no significant relationship between clinical characteristics or DFS and total, small cell size, or large cell size CACs collected one week after surgery (**Table 5** and **Figures 3A–M**).

**Table 5:** Correlation of post-surgery CAC to clinical characteristics (n=35).

Variables	CAC								Small cell size CAC								Large cell size CAC							
	total		Triploidy		Tetraploidy		Multiploidy		total		Triploidy		Tetraploidy		Multiploidy		total		Triploidy		Tetraploidy		Multiploidy	
	+	*p*	+	*p*	+	*p*	+	*p*	+	*p*	+	*p*	+	*p*	+	*p*	+	*p*	+	*p*	+	*p*	+	*p*
**Gender**																								
Male (n=23)	23	0.111	20	0.200	16	0.153	22	0.266	18	0.685	17	0.706	9	0.476	7	0.216	23	0.111	7	1.000	10	0.721	22	0.266
Female (n=12)	10		8		5		10		8		8		3		1		10		3		4		10	
**Age**																								
≤60 (n=10)	10	1.000	8	1.000	5	0.474	10	0.542	9	0.235	8	0.686	4	0.706	3	0.661	10	1.000	1	0.218	2	0.252	10	0.542
>60 (n=25)	24		20		16		22		17		17		8		5		23		9		12		22	
**Smoking history**																								
Never (n=14)	12	0.153	10	0.401	6	0.159	12	0.551	10	1.000	10	1.000	4	0.721	3	1.000	12	0.153	3	0.704	4	0.311	12	0.551
Yes (n=21)	21		18		15		20		16		15		8		5		21		7		10		20	
**Histology**																								
ADC (n=18)	16	0.546	13	0.627	9	0.464	16	1.000	13	1.000	12	0.860	6	0.752	6	0.340	16	0.546	4	0.402	5	0.112	16	1.000
SCC (n=15)	15		13		10		14		11		11		6		2		15		6		7		14	
LCC (n=2)	2		2		2		2		2		2		0		0		2		0		2		2	
**TNM classification**																								
I/II (n=29)	28	0.318	23	1.000	17	1.000	27	0.442	22	0.635	21	1.000	9	0.391	6	0.602	28	0.318	8	1.000	12	1.000	27	0.442
III (n=6)	5		5		4		5		4		4		3		2		5		2		2		5	
**Recurrence**																								
No (n=21)	20	1.000	16	0.676	11	0.311	20	0.551	16	1.000	15	1.000	6	0.477	5	1.000	20	1.000	5	0.474	7	0.483	20	0.551
Yes (n=14)	13		12		10		12		10		10		6		3		13		5		7		12	

These data suggest that in resectable NSCLC before surgery, small cell size CACs, especially of the triploid subtype, are correlated to poor prognosis.

### Variations in CAC Values

**Figure 4:**
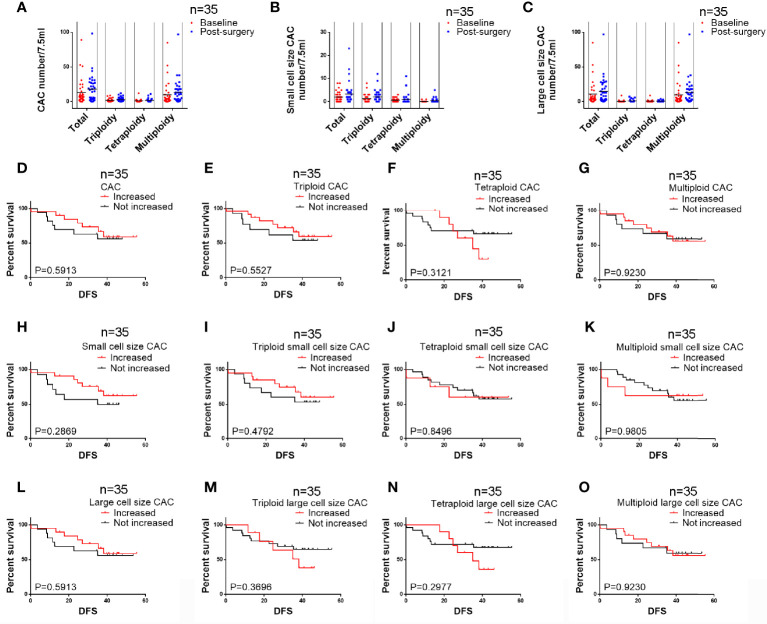
Analysis of CAC pre- and post-surgery variation among 35 patients **(A)** Variation in numbers of total CACs and the three subtypes. **(B)** Variation in numbers of total small cell size CACs and the three subtypes. **(C)** Variation in numbers of total large cell size CACs and the three subtypes. **(D–G)** Variations in total CACs and the triploid, tetraploid, and multiploid subtypes were not correlated to DFS (P > 0.05). **(H–K)** Variations in total small cell size CACs and the triploid, tetraploid, and multiploid small cell size CAC subtypes were not correlated to DFS (P > 0.05). **(L–O)** Variations in total large cell size CACs and the triploid, tetraploid, and multiploid large cell size CAC subtypes were not correlated to DFS (P > 0.05).

We further studied CAC variation in samples derived from 35 patients pre- and post-surgery (454 and 628 CACs, respectively). The average numbers of total, small cell size, and large cell size CACs and their aneuploidy subtypes were slightly increased, with an increase in number from 1 to 5 per patient (**Figures 4A–C** and **Table 6**). Moreover, 54%, 57%, and 54% of patients had more total, small cell size, and large cell size CACs after surgery. For small cell size CACs, the counts of the triploid, tetraploid, and multiploid subtypes increased after surgery in 57%, 23%, and 23% of patients, respectively. For large cell size CACs, the counts of the triploid, tetraploid, and multiploid subtypes increased after surgery in 26%, 29%, and 57% of patients, respectively. Based on the CAC variations pre- and post-surgery, we divided patients into the “Increased” and “Not increased” groups, as shown in **Table 7**. Nevertheless, the variation in number of total, small cell size, and large cell size CACs, as well as all their heteroploid subtypes, was not significantly correlated with clinical features or DFS (**Table 8**) (**Figures 4D–O**) (P>0.05). These data suggest that the variation in post-surgery CAC counts was not associated with tumor recurrence among patients with resectable NSCLC.

**Table 6:** Average CAC number in 35 pairs of patients before and after the surgery.

Variable	CAC		Small cell size CAC		Large cell size CAC	
	Pre-surgery	Post-surgery	Pre-surgery	Post-surgery	Pre-surgery	Post-surgery
	No. of CACs	No. of CACs	No. of CACs	No. of CACs	No. of CACs	No. of CACs
**Monoploid**	0 ± 1	0 ± 0	0 ± 0	0 ± 0	0 ± 0	0 ± 0
**Triploid**	2 ± 2	3 ± 3	1 ± 2	2 ± 3	0 ± 2	1 ± 2
**Tetraploid**	1 ± 2	2 ± 2	1 ± 1	1 ± 2	1 ± 2	1 ± 1
**Multiploid**	10 ± 17	13 ± 18	0 ± 0	0 ± 1	10 ± 17	13 ± 18
**Total**	13 ± 18	18 ± 19	2 ± 2	3 ± 5	11 ± 18	15 ± 19

**Table 7:** CAC variation in the 35 NSCLC cases before and after surgery.

Variations		No. of increased cases	No. of not increased cases
**CAC**	Total	19 (54%)	16 (46%)
	Triploidy	22 (63%)	13 (37%)
	Tetraploidy	11 (31%)	24 (69%)
	Multiploidy	20 (57%)	15 (43%)
**Small cell size CAC**	Total	20 (57%)	15 (43%)
	Triploidy	20 (57%)	15 (43%)
	Tetraploidy	8 (23%)	27 (77%)
	Multiploidy	8 (23%)	27 (77%)
**Large cell size CAC**	Total	19 (54%)	16 (46%)
	Triploidy	9 (26%)	26 (74%)
	Tetraploidy	10 (29%)	25 (71%)
	Multiploidy	20 (57%)	15 (43%)

**Table 8:** Correlations between clinical characteristics and CAC variations pre- and post-surgery in 35 NSCLC patients.

Variables	CAC								Small cell size CAC								Large cell size CAC							
	total		Triploidy		Tetraploidy		Multiploidy		total		Triploidy		Tetraploidy		Multiploidy		total		Triploidy		Tetraploidy		Multiploidy	
	IG	*p*	IG	*p*	IG	*p*	IG	*p*	IG	*p*	IG	*p*	IG	*p*	IG	*p*	IG	*p*	IG	*p*	IG	*p*	IG	*p*
**Gender**																								
Male (n=23)	10	0.152	14	1.000	8	1.000	12	0.489	13	1.000	12	0.489	7	0.216	7	0.216	11	0.476	7	0.450	7	1.000	12	0.489
Female (n=12)	9		8		3		8		7		8		1		1		8		2		3		8	
**Age**																								
≤60 (n=10)	3	0.132	5	0.444	1	0.120	5	0.712	4	0.266	5	0.712	1	0.390	3	0.661	4	0.454	1	0.235	1	0.218	5	0.712
>60 (n=25)	16		17		10		15		16		15		7		5		15		8		9		15	
**Smoking history**																								
Never (n=14)	9	0.491	10	0.488	3	1.000	8	1.000	9	0.728	10	0.296	2	0.431	3	1.000	8	1.000	2	0.262	2	0.252	8	1.000
Yes (n=21)	10		12		8		12		11		10		6		5		11		7		8		12	
**Histology**																								
ADC (n=18)	10	1.000	11	1.000	5	0.177	12	0.488	12	0.488	12	0.488	4	1.000	6	0.340	11	0.741	4	0.834	4	0.105	12	0.488
SCC (n=15)	8		10		4		7		7		7		4		2		7		5		4		7	
LCC (n=2)	1		1		2		1		1		1		0		0		1		0		2		1	
**TNM classification**																								
I/II (n=29)	15	0.666	18	1.000	9	1.000	16	0.680	18	0.367	17	1.000	7	1.000	6	0.602	15	0.666	7	0.635	8	1.000	16	0.680
III (n=6)	4		4		2		4		2		3		1		2		4		2		2		4	
**Recurrence**																								
No (n=21)	12	0.739	14	0.724	5	0.283	12	1.000	13	0.511	13	0.511	5	1.000	5	1.000	12	0.739	4	0.432	4	0.151	12	1.000
Yes (n=14)	7		8		6		8		7		7		3		3		7		5		6		8	

IG: case number of “increased group” after surgery.

## Discussion

The detection of CTCs to monitor the prognosis of cancer patients has been previously reported (34, 35). Based on the pervasiveness of aneuploidy in solid tumors, we used the SE-iFISH system to detect chromosome 8 ploidy, identifying CACs in 50 patients with stage I to IIIA NSCLC who underwent surgery. In this study, we investigated a variety of correlations between clinical significance and karyotypic subtype or CAC size.

The CellSearch system has been approved for the detection of CTCs as a novel clinical marker and prognostic factor in metastasized breast, colorectal, prostate, and lung cancers (14, 17, 36, 37). However, the incidence of CTCs in NSCLC was reportedly lower than that in other cancers such as prostate, breast, ovarian, and colorectal cancer, as determined using the EpCAM-dependent method (38). Lin’s previous study validated the SE-iFISH method with over 80% recovery efficiency in lung cancer cells (20), and no CAC was found in 30 healthy donors (29). Previous studies using the conventional CellSearch method reported positive rates of only 23%–36% among NSCLC patients (37, 39, 40).By contrast, the SE-iFISH method used in this study previously yielded a 92% (24/26) CTC-positive rate among lung cancer patients (20). Similarly, Ye et al. evaluated CTCs in 594 blood samples from 19 various carcinomas, suggesting that the overall positive rate of CTCs in 594 samples was 89.0%, with the CTC-positive rate of lung cancer being 92.9% (79/85) through the use of SE-iFISH (29). Consistent with these data, we found that 88% and 94% of patients were positive for total CACs pre- and post-surgery, respectively, although total CACs did not significantly correlate with DFS in resectable NSCLC.

In addition to the high CAC-positive rate in NSCLC detected using the SE-iFISH method, we then wondered whether all the CACs were malignant cells. Therefore, we divided CACs into small cell size and large cell size groups. It was shown that small cell size CACs (≤ 5 µm) accounted for only a small proportion of total CACs (≤20%), whereas large cell size CACs (>5 µm) were the majority (≥80%) among the 50 cases. However, small cell size CACs, but not large cell size CACs, were related to poor outcomes in resectable NSCLC patients in our study. A previous study of advanced lung cancer showed that small cell size CTCs accounted for 52.8% of the absolute CTC number and were related to progression-free survival (40). Evidently, small cell size CACs were significantly fewer in resectable NSCLC cases than in advanced NSCLC. Another study suggested that tumor cells undergoing EMT were smaller in size than those without EMT characteristics (41) and that EMT is generally regarded as the reason for cancer relapse, metastasis, and poor prognosis (19). Wang et al. further showed that the majority of Vimentin^+^ (a marker for mesenchymal cells) CACs were small in size and completely different from Vimentin^−^ cells (32). All of these studies suggest that small cell size CACs with more malignant behavior are closely related to tumor progression and poor prognosis.

Aneuploidy, which can cause tumor formation, remains the most common feature of chromosomal instability in solid cancer (25). Coward et al. further explained this by suggesting that polyploid tumor cells, with significantly elevated genomic content, facilitate rapid tumor evolution and the acquisition of therapy resistance (26). A previous study clarified that SE-iFISH was feasible for efficient co-detection and *in situ* phenotypic and karyotypic characterization, as well as for quantification of CACs, which made further classification into diverse subtypes possible based on chromosome ploidy and biomarker expression (30). Numerous papers have already described the clinical significance of aneuploid CACs (15, 27, 30, 33, 42, 43). Ye et al. even studied 594 blood samples from 19 different carcinomas using the SE-iFISH method and found that patients at stage III–IV had more tetraploid, polyploid, large cell size, and total CTCs compared with those in patients at stage I–II (29). In addition, the latest research in 18 healthy volunteers and 34 early‐stage and 24 advanced lung adenocarcinoma patients suggested that combined detection of specific aneuploid subtypes of circulating tumor endothelial cells and CTCs may facilitate diagnosis in early-stage patients with a higher sensitivity and specificity (15).Therefore, we focused on changes in the different CAC ploidies before and after surgery. We classified CACs into the karyotypic triploid, tetraploid, and multiploid subtypes. We found that small cell size CACs had a higher percentage of triploidy, whereas large cell size CACs possessed a higher multiploidy ratio. Triploid CACs and triploid small cell size CACs were related to shorter DFS and poor clinical features. We reviewed related articles from PubMed and found that the triploid CTC subtype is considered to be related to therapeutic resistance in several solid tumors. Chen et al. found that in patients with esophageal cancer, those without triploid cells were more sensitive to chemotherapy than those with them (33). Similarly, Li et al. also suggested that an increased percentage of triploid CTCs was associated with chemotherapy resistance in advanced gastric cancer (27). As for pancreatic cancer, Xu et al. found that triploid CTC number could not only predict chemo-sensitivity but was also associated with reduced 1-year survival (43). Our study, focusing on 3-year DFS in resectable NSCLC, further suggested that triploid small cell size CACs, not triploid large cell size CACs, might be more helpful for predicting prognosis. The above data may suggest that triploid cells are more malignant and can hence lead to proliferation, progression, and chemotherapy resistance. Triploid CACs or triploid small cell size CACs may have potential as new prognostic predictors and therapeutic targets in tumors, including NSCLC.

We also investigated how CAC number and CAC ploidy changed before and after surgical treatment. After resection, the CAC number and subtypes increased slightly, but no significant correlation was found between DFS and the variation pre- and post-surgery. Treatment through surgery may facilitate the entry of CACs into the general circulation, and these cells were mostly nonmalignant CACs or endothelial cells, which could be gradually eliminated by circulating immune cells (44–46). Among patients who underwent breast cancer resection, the CAC number was reportedly elevated 3–4 days after surgery but fell back to the pre-surgery condition one week post-surgery (47). However, no publication has yet illustrated the proper time for post-surgery CTC or CAC blood collection in lung cancer. In our study, CACs kept increasing among patients with resectable NSCLC one week after surgery, suggesting that blood collection may need to be performed much later. A previous study illustrated that an operation was not able to reduce CTCs in all cases, and a considerable number of CTCs remained in circulation following resection of the primary tumor. Moreover, some CTCs may already be pre-existing in the patients’ circulation, and not derived from the resection of primary malignant tumors, influencing later metastasis and recurrence (34, 48, 49). This may be why post-surgery CACs had a weak influence on clinical outcome in our study.

## Conclusions

In summary, we used SE-iFISH to detect CACs in patients with resectable NSCLC. We first divided total CACs into small and large sizes and further classified these CACs into three heteroploid subtypes based on chromosome 8 ploidy. We found that small cell size CACs accounted for no more than 20% of total CACs and were significantly related to shorter DFS in patients with resectable NSCLC. Pre-surgery small cell size CACs, especially of the triploid subtype, were significantly correlated with later TNM stage and recurrence, suggesting that this subtype might be a good biomarker for poor prognosis in patients with resectable NSCLC.

## Data Availability Statement

The raw data supporting the conclusions of this article will be made available by the authors, without undue reservation.

## Ethics Statement

The studies involving human participants were reviewed and approved by the medical ethics committee of Beijing Cancer Hospital. The patients/participants provided their written informed consent to participate in this study.

## Author Contributions

YH, YM, and CL contributed to conception and design of the study. Medical practitioners YY, CL, JieZ, YX, JiaZ, and JF provided patients and patient data for the study. YH, YM, and CL organized the database, and YH performed the statistical analysis. JS supported the literature and statistical methods. PL supported the detection method. YH wrote the manuscript. All authors contributed to the article and approved the submitted version.

## Funding

This work was supported by National Key R&D Program of China (grant number 2018YFC0910700), National Natural Science Foundation of China (grant number 81772494 and 81502578), Science Foundation of Peking University Cancer Hospital 2020-2, Capital’s Funds for Health Improvement and Research (grant number 2020-2-2153), and Beijing Nova Program (Z201100006820092) from Beijing Municipal Science & Technology Commission

## Conflict of Interest

Author PPL was employed by Cytelligen, San Diego, CA.

The remaining authors declare that the research was conducted in the absence of any commercial or financial relationships that could be construed as a potential conflict of interest.

## References

[1] Siegel R Naishadham D Jemal A. Cancer statistics, 2013. CA Cancer J Clin (2013) 63(1):11–30. 10.3322/caac.21166 23335087

[2] Torre LA Siegel RL Jemal A. Lung Cancer Statistics. Adv Exp Med Biol (2016) 893:1–19. 10.1007/978-3-319-24223-1_1 26667336

[3] Goldstraw P Crowley J Chansky K Giroux DJ Groome PA Rami-Porta R. The IASLC Lung Cancer Staging Project: proposals for the revision of the TNM stage groupings in the forthcoming (seventh) edition of the TNM Classification of malignant tumours. J Thorac Oncol (2007) 2(8):706–14. 10.1097/JTO.0b013e31812f3c1a 17762336

[4] Ramalingam SS Owonikoko TK Khuri FR. Lung cancer: New biological insights and recent therapeutic advances. CA Cancer J Clin (2011) 61(2):91–112. 10.3322/caac.20102 21303969

[5] Yano T Hara N Ichinose Y Asoh H Yokoyama H Ohta M. Local recurrence after complete resection for non-small-cell carcinoma of the lung. Significance of local control by radiation treatment. J Thorac Cardiovasc Surg (1994) 107(1):8–12. 10.1016/S0022-5223(94)70445-7 8283923

[6] Pantel K. Blood-Based Analysis of Circulating Cell-Free DNA and Tumor Cells for Early Cancer Detection. PloS Med (2016) 13(12):e1002205. 10.1371/journal.pmed.1002205 28027295 PMC5189941

[7] Najjar F Alammar M Al-Massarani G Almalla N Japawe A Ikhtiar A. Circulating endothelial cells and microparticles as diagnostic and prognostic biomarkers in small-cell lung cancer. Lung Cancer (2018) 124:23–30. 10.1016/j.lungcan.2018.06.033 30268466

[8] Ren S Ren XD Guo LF Qu XM Shang MY Dai XT. Urine cell-free DNA as a promising biomarker for early detection of non-small cell lung cancer. J Clin Lab Anal (2020) 13:e23321. 10.1002/jcla.23321 PMC7439414 32281142

[9] Patnaik SK Cortes EG Kannisto ED Punnanitinont A Dhillon SS Liu S. Lower airway bacterial microbiome may influence recurrence after resection of early-stage non-small cell lung cancer. J Thorac Cardiovasc Surg (2021) 161(2):419–29.e16. 10.1016/j.jtcvs.2020.01.104 32340803

[10] de Miguel-Perez D Bayarri-Lara CI Ortega FG Russo A Moyano Rodriguez MJ Alvarez-Cubero MJ. Post-Surgery Circulating Tumor Cells and AXL Overexpression as New Poor Prognostic Biomarkers in Resected Lung Adenocarcinoma. Cancers (Basel) (2019) 11(11):1750. 10.3390/cancers11111750 PMC6896005 31703465

[11] Herreros-Pomares A de-Maya-Girones JD Calabuig-Fariñas S Lucas R Martínez A Pardo-Sánchez JM. Lung tumorspheres reveal cancer stem cell-like properties and a score with prognostic impact in resected non-small-cell lung cancer. Cell Death Dis (2019) 10(9):660. 10.1038/s41419-019-1898-1 31506430 PMC6737160

[12] Ma G Jiang Y Liang M Li J Wang J Mao X. Dynamic monitoring of CD45-/CD31+/DAPI+ circulating endothelial cells aneuploid for chromosome 8 during neoadjuvant chemotherapy in locally advanced breast cancer. Ther Adv Med Oncol (2020) 12:1758835920918470. 10.1177/1758835920918470 32489429 PMC7238307

[13] Netterberg I Karlsson MO Terstappen L Koopman M Punt CJA Friberg LE. Comparing Circulating Tumor Cell Counts with Dynamic Tumor Size Changes as Predictor of Overall Survival: A Quantitative Modeling Framework. Clin Cancer Res (2020) 26(18):4892–900. 10.1158/1078-0432.CCR-19-2570 32527941

[14] Cieslikowski WA Budna-Tukan J Swierczewska M Ida A Hrab M Jankowiak A. Circulating Tumor Cells as a Marker of Disseminated Disease in Patients with Newly Diagnosed High-Risk Prostate Cancer. Cancers (Basel) (2020) 12(1):160. 10.3390/cancers12010160 PMC7017349 31936460

[15] Lei Y Sun N Zhang G Liu C Lu Z Huang J. Combined detection of aneuploid circulating tumor-derived endothelial cells and circulating tumor cells may improve diagnosis of early stage non-small-cell lung cancer. Clin Transl Med (2020) 10(3):e128. 10.1002/ctm2.128 32659050 PMC7418803

[16] Syrigos K Fiste O Charpidou A Grapsa D. Circulating tumor cells count as a predictor of survival in lung cancer. Crit Rev Oncol Hematol (2018) 125:60–8. 10.1016/j.critrevonc.2018.03.004 29650278

[17] Goodman C Speers CW. The role of circulating tumor cells in breast cancer and implications for radiation treatment decisions. Int J Radiat Oncol Biol Phys (2020) 109(1):44–59. 10.1016/j.ijrobp.2020.08.039 32882354

[18] Yu M Stott S Toner M Maheswaran S Haber DA. Circulating tumor cells: approaches to isolation and characterization. J Cell Biol (2011) 192(3):373–82. 10.1083/jcb.201010021 PMC3101098 21300848

[19] Genna A Vanwynsberghe AM Villard AV Pottier C Ancel J Polette M. EMT-Associated Heterogeneity in Circulating Tumor Cells: Sticky Friends on the Road to Metastasis. Cancers (Basel) (2020) 12(6):1632. 10.3390/cancers12061632 PMC7352430 32575608

[20] Ge F Zhang H Wang DD Li L Lin PP. Enhanced detection and comprehensive in situ phenotypic characterization of circulating and disseminated heteroploid epithelial and glioma tumor cells. Oncotarget (2015) 6(29):27049–64. 10.18632/oncotarget.4819 PMC4694973 26267323

[21] Pailler E Auger N Lindsay CR Vielh P Islas-Morris-Hernandez A Borget I. High level of chromosomal instability in circulating tumor cells of ROS1-rearranged non-small-cell lung cancer. Ann Oncol (2015) 26(7):1408–15. 10.1093/annonc/mdv165 PMC4478971 25846554

[22] Hofman V Ilie M Long E Guibert N Selva E Washetine K. Detection of circulating tumor cells from lung cancer patients in the era of targeted therapy: promises, drawbacks and pitfalls. Curr Mol Med (2014) 14(4):440–56. 10.2174/1566524014666140414205455 24730524

[23] Weaver BA Cleveland DW. Does aneuploidy cause cancer? Curr Opin Cell Biol (2006) 18(6):658–67. 10.1016/j.ceb.2006.10.002 17046232

[24] Gordon DJ Resio B Pellman D. Causes and consequences of aneuploidy in cancer. Nat Rev Genet (2012) 13(3):189–203. 10.1038/nrg3123 22269907

[25] Kops GJ Weaver BA Cleveland DW. On the road to cancer: aneuploidy and the mitotic checkpoint. Nat Rev Cancer (2005) 5(10):773–85. 10.1038/nrc1714 16195750

[26] Coward J Harding A. Size Does Matter: Why Polyploid Tumor Cells are Critical Drug Targets in the War on Cancer. Front Oncol (2014) 4:123. 10.3389/fonc.2014.00123 24904834 PMC4033620

[27] Li Y Zhang X Ge S Gao J Gong J Lu M. Clinical significance of phenotyping and karyotyping of circulating tumor cells in patients with advanced gastric cancer. Oncotarget (2014) 5(16):6594–602. 10.18632/oncotarget.2175 PMC4196148 25026283

[28] Lin PP. Integrated EpCAM-independent subtraction enrichment and iFISH strategies to detect and classify disseminated and circulating tumors cells. Clin Transl Med (2015) 4(1):38. 10.1186/s40169-015-0081-2 26718583 PMC4696935

[29] Ye Z Ding Y Chen Z Li Z Ma S Xu Z. Detecting and phenotyping of aneuploid circulating tumor cells in patients with various malignancies. Cancer Biol Ther (2019) 20(4):546–51. 10.1080/15384047.2018.1538000 PMC6422472 30572767

[30] Lin PP Gires O Wang DD Li L Wang H. Comprehensive in situ co-detection of aneuploid circulating endothelial and tumor cells. Sci Rep (2017) 7(1):9789. 10.1038/s41598-017-10763-7 28852197 PMC5575124

[31] Gao Y Zhu Y Zhang Z Zhang C Huang X Yuan Z. Clinical significance of pancreatic circulating tumor cells using combined negative enrichment and immunostaining-fluorescence in situ hybridization. J Exp Clin Cancer Res (2016) 35:66. 10.1186/s13046-016-0340-0 27066900 PMC4828870

[32] Wang Y Liu Y Zhang L Tong L Gao Y Hu F. Vimentin expression in circulating tumor cells (CTCs) associated with liver metastases predicts poor progression-free survival in patients with advanced lung cancer. J Cancer Res Clin Oncol (2019) 145(12):2911–20. 10.1007/s00432-019-03040-9 PMC6861204 31646374

[33] Chen Y Yang Z Wang Y Wang J Wang C. Karyotyping of circulating tumor cells for predicting chemotherapeutic sensitivity and efficacy in patients with esophageal cancer. BMC Cancer (2019) 19(1):651. 10.1186/s12885-019-5850-7 31269908 PMC6609398

[34] Bayarri-Lara C Ortega FG Cueto Ladron de Guevara A Puche JL Ruiz Zafra J de Miguel-Perez D. Circulating Tumor Cells Identify Early Recurrence in Patients with Non-Small Cell Lung Cancer Undergoing Radical Resection. PloS One (2016) 11(2):e0148659. 10.1371/journal.pone.0148659 26913536 PMC4767413

[35] Martini V Timme-Bronsert S Fichtner-Feigl S Hoeppner J Kulemann B. Circulating Tumor Cells in Pancreatic Cancer: Current Perspectives. Cancers (Basel) (2019) 11(11):1659. 10.3390/cancers11111659 PMC6895979 31717773

[36] Arrazubi V Mata E Antelo ML Tarifa A Herrera J Zazpe C. Circulating Tumor Cells in Patients Undergoing Resection of Colorectal Cancer Liver Metastases. Clinical Utility for Long-Term Outcome: A Prospective Trial. Ann Surg Oncol (2019) 26(9):2805–11. 10.1245/s10434-019-07503-8 31209673

[37] Krebs MG Sloane R Priest L Lancashire L Hou JM Greystoke A. Evaluation and prognostic significance of circulating tumor cells in patients with non-small-cell lung cancer. J Clin Oncol (2011) 29(12):1556–63. 10.1200/JCO.2010.28.7045 21422424

[38] Allard WJ Matera J Miller MC Repollet M Connelly MC Rao C. Tumor cells circulate in the peripheral blood of all major carcinomas but not in healthy subjects or patients with nonmalignant diseases. Clin Cancer Res (2004) 10(20):6897–904. 10.1158/1078-0432.CCR-04-0378 15501967

[39] Hirose T Murata Y Oki Y Sugiyama T Kusumoto S Ishida H. Relationship of circulating tumor cells to the effectiveness of cytotoxic chemotherapy in patients with metastatic non-small-cell lung cancer. Oncol Res (2012) 20(2-3):131–7. 10.3727/096504012X13473664562583 23193919

[40] Juan O Vidal J Gisbert R Munoz J Macia S Gomez-Codina J. Prognostic significance of circulating tumor cells in advanced non-small cell lung cancer patients treated with docetaxel and gemcitabine. Clin Transl Oncol (2014) 16(7):637–43. 10.1007/s12094-013-1128-8 24217975

[41] Ito H Inoue H Kimura S Ohmori T Ishikawa F Gohda K. Prognostic impact of the number of viable circulating cells with high telomerase activity in gastric cancer patients: a prospective study. Int J Oncol (2014) 45(1):227–34. 10.3892/ijo.2014.2409 24788213

[42] Li Y Zhang X Liu D Gong J Wang DD Li S. Evolutionary Expression of HER2 Conferred by Chromosome Aneuploidy on Circulating Gastric Cancer Cells Contributes to Developing Targeted and Chemotherapeutic Resistance. Clin Cancer Res (2018) 24(21):5261–71. 10.1158/1078-0432.CCR-18-1205 30012565

[43] Xu Y Qin T Li J Wang X Gao C Xu C. Detection of Circulating Tumor Cells Using Negative Enrichment Immunofluorescence and an In Situ Hybridization System in Pancreatic Cancer. Int J Mol Sci (2017) 18(4):622. 10.3390/ijms18040622 PMC5412265 28333072

[44] Glaves D. Correlation between circulating cancer cells and incidence of metastases. Br J Cancer (1983) 48(5):665–73. 10.1038/bjc.1983.248 PMC2011528 6639858

[45] Lv C Zhao B Wang L Zhang P Ma Y Wang Y. Detection of circulating tumor cells in pulmonary venous blood for resectable non-small cell lung cancer. Oncol Lett (2018) 15(1):1103–12. 10.3892/ol.2017.7405 PMC5772954 29422972

[46] Leone K Poggiana C Zamarchi R. The Interplay between Circulating Tumor Cells and the Immune System: From Immune Escape to Cancer Immunotherapy. Diagnost (Basel) (2018) 8(3):59. 10.3390/diagnostics8030059 PMC6164896 30200242

[47] Zhang Y Lv Y Niu Y Su H Feng A. Role of Circulating Tumor Cell (CTC) Monitoring in Evaluating Prognosis of Triple-Negative Breast Cancer Patients in China. Med Sci Monit (2017) 23:3071–9. 10.12659/MSM.902637 PMC5493060 28643770

[48] Wang L Li Y Xu J Zhang A Wang X Tang R. Quantified postsurgical small cell size CTCs and EpCAM(+) circulating tumor stem cells with cytogenetic abnormalities in hepatocellular carcinoma patients determine cancer relapse. Cancer Lett (2018) 412:99–107. 10.1016/j.canlet.2017.10.004 29031565

[49] Sandri MT Zorzino L Cassatella MC Bassi F Luini A Casadio C. Changes in circulating tumor cell detection in patients with localized breast cancer before and after surgery. Ann Surg Oncol (2010) 17(6):1539–45. 10.1245/s10434-010-0918-2 20135356

